# Reference Database for a Novel Binocular Visual Function Perimeter: A Randomized Clinical Trial

**DOI:** 10.1016/j.xops.2024.100583

**Published:** 2024-07-20

**Authors:** Vincent Michael Patella, Nevin W. El-Nimri, John G. Flanagan, Mary K. Durbin, Timothy Bossie, Derek Y. Ho, Mayra Tafreshi, Michael A. Chaglasian, David Kasanoff, Satoshi Inoue, Sasan Moghimi, Takashi Nishida, Murray Fingeret, Robert N. Weinreb

**Affiliations:** 1 Department of Ophthalmology, University of Iowa Health Care, Iowa City, Iowa; 2 Topcon Healthcare, Oakland, New Jersey; 3 Herbert Wertheim School of Optometry & Vision Science, University of California Berkeley, Berkeley, California; 4 New England College of Optometry, Boston, Massachusetts; 5 Illinois College of Optometry, Chicago, Illinois; 6 New View Optometric Center, La Mesa, California; 7 CREWT Medical Systems, Inc, Tokyo, Japan; 8 Viterbi Family Department of Ophthalmology, Hamilton Glaucoma Center, Shiley Eye Institute, San Diego, California; 9 SUNY College of Optometry, New York, New York

**Keywords:** 10-degree field, 24-degree field, Perimetry, Reference database, Visual field

## Abstract

**Purpose:**

To construct a comprehensive reference database (RDB) for a novel binocular automated perimeter.

**Design:**

A four-site prospective randomized clinical trial.

**Subjects and Controls:**

Three hundred fifty-six healthy subjects without ocular conditions that might affect visual function were categorized into 7 age groups.

**Methods:**

Subjects underwent comprehensive ocular examination of both eyes before enrollment. Using the TEMPO/IMOvifa automated perimeter (Topcon Healthcare/CREWT Medical Systems), each subject completed 4 binocular threshold visual field (VF) tests during a single visit: First, practice 24-2 and 10-2 tests were obtained from both eyes. Next, study 24-2 and 10-2 tests were obtained from both eyes. Test order of each sequence was randomized, and the tests were conducted under standard automated perimetry testing conditions: Goldmann stimulus size III, 3183 cd/m^2^ maximum stimulus intensity, and background intensity of 10 cd/m^2^, using AIZE-Rapid test strategy. Standard VF reliability indices were assessed. For each subject, 24-2 and 10-2 test results from 1 randomly selected eye were analyzed.

**Main Outcome Measures:**

Perimetric threshold sensitivity and reference limits for each test analysis parameter.

**Results:**

The ages of the study cohort were widely distributed, with a mean age (standard deviation [SD]) of 52.3 (18.5) years. Sex assignment was 44.0% male and 56.0% female. The majority of subjects self-identified as White (67.4%), followed by Black or African American (13.5%) and Asian (8.7%), with 14.6% self-identified as Hispanic or Latino ethnicity. Mean sensitivity (SD) was 29.1 (1.3) decibels (dB) for the 24-2 and 32.4 (1.0) dB for the 10-2 test. For the 24-2 and 10-2, mean sensitivity (SD) age-related changes averaged −0.06 (0.01) dB and −0.05 (0.01) dB per year, respectively. The normal range of pointwise threshold sensitivity increased with eccentricity and showed asymmetry around the mean, particularly notable in the 24-2 test. Mean (SD) binocular test duration was 3.18 (0.38) minutes (1 minute 35 seconds per eye) for the 24-2 test and 3.58 (0.43) minutes (1 minute 47 seconds per eye) for the 10-2 test.

**Conclusions:**

An RDB for the TEMPO/IMOvifa perimeter was established, highlighting the significance of considering both age and stimulus eccentricity in interpreting threshold VF test results.

**Financial Disclosure(s):**

Proprietary or commercial disclosure may be found in the Footnotes and Disclosures at the end of this article.

Automated static threshold perimetry continues to have a central role in the diagnosis and management of various eye diseases, especially glaucoma.^1^^,^^2^ Analysis of visual field (VF) test results is commonly based upon comparisons to empirically determined age-corrected ranges of peripheral and foveal visual sensitivity found at specific test point locations in healthy subjects. In aggregate, these age-corrected ranges are commonly referred to as a perimetric reference database (RDB). The statistical distribution of this reference data is determined using conventional statistical methods and is known to vary among perimetric devices and testing strategies, due to design differences.^3^, ^4^, ^5^, ^6^, ^7^

The concept of RDBs was introduced to eye care in 1958, when Leydhecker et al published tonometric measurements from a mass screening of German subjects, thus establishing a reference range for intraocular pressure that is still in use today.^8^ The first multicenter perimetric RDB was developed in 1986 by Heijl et al in the form of a software package for the Humphrey perimeter.^4^ The perimeter’s RDB was based upon the results of 487 tests of 239 healthy subjects conducted at 4 sites, 1 in Sweden and 3 in the United States. Along with subsequent enhancements, it significantly improved the ability of clinicians to identify early and subtle VF defects that might have been otherwise overlooked.^7^ In the present era, the methods of Heijl et al remain the accepted standard for perimetric RDB design.

The TEMPO/IMOvifa perimeter (Topcon Healthcare/CREWT Medical Systems) is an automated device that conducts simultaneous VF testing of both eyes, under commonly accepted examination conditions, generally referred to as standard automated perimetry (SAP).^9^, ^10^, ^11^ The purpose of this study was to develop a comprehensive age-corrected RDB for the TEMPO perimeter.

## Methods

### Approval and Ethical Considerations

The study protocol received approval from the Advarra Institutional Review Board (Columbia, MD, United States) on February 15, 2023 (IRB approval Pro00069562). The research adhered to the principles of the Declaration of Helsinki and the Health Insurance Portability and Accountability Act. Verbal and written informed consent were obtained from all study subjects.

The registration information for this human clinical trial is available to the public at http://www.clinicaltrials.gov (identifier: NCT05792046) (Supplementary Figure 1, available at www.ophthalmologyscience.org).

### Study Population

A prospective randomized clinical trial across 4 sites in the United States included 356 healthy subjects who did not have any ocular condition known to affect visual function. Subjects were categorized into 7 age groups (22–29, 30–39, 40–49, 50–59, 60–69, 70–79, and ≥80 years), with the intention that study subjects should be approximately evenly distributed across the age groups.

Subjects were recruited at the time of regularly scheduled eye examinations at each of the clinical sites, and by way of referrals from friends and family, and public flyers.

Study inclusion criteria required the following: age ≥22 years, refractively corrected visual acuity 20/40 or better, intraocular pressure ≤21 mmHg for both eyes, and absence of ocular conditions known to impact visual function. Subjects with a history of ocular conditions adversely affecting visual function, those unable to tolerate ophthalmic imaging or VF testing, subjects with a spherical equivalent refractive error exceeding ±6 diopters, and those with a cylindrical refractive error exceeding ±2.5 diopters were excluded from the study.

The 4 study sites were all in the United States: the New England College of Optometry, Boston, MA, the Illinois College of Optometry, Chicago, IL, the New View Optometric Center, La Mesa, CA, and Topcon Healthcare Innovation Center, San Diego, CA.

### Study Visit(s)

All subjects underwent clinical examination of both eyes within 6 months prior to entry into the trial to confirm eligibility requirements. Eye examination included subjective refraction and determination of best corrected visual acuity, pupillary responses, extraocular muscle assessment, slit-lamp biomicroscopy of the anterior segment, intraocular pressure measurement, and examination of the posterior segment. Visual field testing was not included in this examination, nor was having a normal VF a requirement for inclusion as a subject.^12^

Maestro2 OCT (Topcon Healthcare) 12 mm × 9 mm widefield scans and fundus photos of both eyes were captured during the screening visit but were not a condition for enrollment.

### VF Testing

Subjects were tested binocularly during a single visit using the TEMPO perimeter (Topcon Healthcare/CREWT Medical Systems). In cases where binocular testing was not possible, subjects were tested 1 eye at a time. The inability to perform binocular testing may arise from subjects perceiving diplopia during the VF test, experiencing difficulty in fusion, eyes not aligning with the centers of the binocular optics in the VF test, or encountering any other condition(s) (e.g., strabismus, amblyopia, anisometropia, nystagmus) hindering the binocular testing process. For each subject, a single eye was randomly chosen for inclusion in the RDB.

Each subject completed a series of 4 VF examinations of each eye, comprising practice 24-2 and 10-2 tests, followed by study 24-2 and 10-2 tests of both eyes. Test order of each sequence was randomized. These tests were conducted using fixed perimetric settings: Goldmann stimulus size III, AIZE-Rapid test strategy, foveal threshold testing, 3183 cd/m^2^ maximum stimulus intensity, background intensity of 10 cd/m^2^, stimulus duration of 200 milliseconds, and Heijl-Krakau blind spot gaze stability assessment. The TEMPO/IMOvifa perimeter features 2 independent optical systems that display stimuli to each eye independently.^13^^,^^14^

Visual field reliability indices were assessed, and subjects were reinstructed and retested if unacceptable reliability findings were obtained in either eye (>25% in any of the following: fixation losses, false positive errors, or false negative errors).^15^ All subjects were allowed up to 3 attempts to produce VF tests meeting reliability index requirements.

A data committee consisting of 2 experienced VF experts (V.M.P. and M.F.) reviewed all VF test results. They confirmed that each test met the inclusion criteria and identified VFs that appeared questionable, which upon further investigation, sometimes led to the discovery of abnormal eye conditions that had been overlooked in the clinical examination.

### Statistical Analysis

Descriptive statistics (number of samples, mean, standard deviation [SD], median, minimum, and maximum) and 95% confidence interval (CI) were reported. Age-correction coefficients for perimetric threshold sensitivity were calculated cross sectionally at each test point by linear regression. Reference limits for each test analysis parameter (mean deviation, pattern SD, total deviation, pattern deviation, and foveal threshold) were calculated at the following levels: 5%, 2%, 1%, and 0.5%.^11^^,^^13^

Because no large datasets of TEMPO exist yet, the sample size was determined using publicly available VF data from normal subjects (the State University of New York-Indiana University dataset of healthy eyes with 263 values for 24-2 static automated perimetry parameters).^16^^,^^17^ The goal was to collect enough data so that nonparametric estimates of the 0.5th, first, second, and fifth percentiles of the VF parameters would seldom have overlapping CIs. The State University of New York-Indiana University dataset was replicated 3 times, resulting in 789 values. We then applied random sampling with replacement to create 1000 simulated datasets with n values for different VF parameters. Nonparametric estimations of the 95% CIs for the 0.5th, first, second, and fifth percentiles of the VF parameters without any covariate adjustments were performed. The point-wise parameters, total deviation, and pattern deviation showed no overlap between the 95% CIs for the 0.5th, first, second, and fifth percentiles in >85% of the simulations when n = 356, so this sample size was deemed sufficient.

## Results

The study cohort displayed a diverse age distribution, with the highest proportion of subjects falling in the 60- to 69-year age range (17.4%) and the lowest proportion among those aged ≥80 years (6.5%). Participants had a mean (SD) age of 52.3 (18.5) years. The sex distribution was 44.0% male and 56.0% female. The majority of subjects self-identified as White (67.4%), followed by Black or African American (13.5%) and Asian (8.7%); 14.6% of participants self-identified as Hispanic or Latino (Table 1).

**Table 1:** Patient Demographic and Clinical Characteristics

Demographics	Total (n) = 356
Age (SD)	52.3 (18.5)
Age distribution	
22-29	59 (16.6%)
30-39	51 (14.3%)
40-49	56 (15.7%)
50-59	52 (14.6%)
60-69	62 (17.4%)
70-79	53 (14.9%)
>80	23 (6.5%)
Sex	
Male	157 (44.0%)
Female	199 (56.0%)
Race	
White	240 (67.4%)
Black or African American	48 (13.5%)
Asian	31 (8.7%)
Native American or Alaska Native	1 (0.3%)
Native Hawaiian or Other Pacific Islander	3 (0.8%)
2+ races	17 (4.8%)
Other	16 (4.5%)
Ethnicity	
Hispanic or Latino	52 (14.6%)
Not Hispanic or Latino	304 (85.4%)
Eye characteristics, mean (SD)	
Right/left	174 (48.9%)/182 (51.1%)
24-2 foveal threshold, dB	34.1 (4.3)
24-2 MS, dB	29.1 (1.3)
10-2 foveal threshold, dB	34.0 (4.7)
10-2 MS, dB	32.4 (1.0)

dB = decibels; MS = mean sensitivity; SD = standard deviation.

Recruitment began in March 2023 and concluded in August 2023. Across all sites, a total of 376 subjects were initially enrolled (Table 2). A total of 20 subjects were excluded for the reasons detailed in Table 2, resulting in the inclusion of 356 subjects. Four of the 20 subjects were excluded due to retinal abnormalities identified in retinal photographs by the data committee. Of the 356 included subjects, 15 were unable to perform binocular perimetric testing and instead underwent monocular testing. There were no subjects excluded or tests repeated due to failing to meet the VF reliability index criteria. There were no adverse events during the study.

**Table 2:** An Overview of Excluded Subjects, Specifying the Reasons for Exclusion, Along With the Corresponding Total Counts of Enrolled, Excluded, and Included Subjects at Each Site

**Site**	Enrolled Subjects	Exclusion Reasons						Total Numbers	
		Ocular condition affecting VF	RE outside range	Study eye not eligible per data committee	Did not complete all study requirements	Unable to tolerate ophthalmic imaging	IOP ≥22	Excluded subjects	Included subjects
1	128	5	2	1			1	9	119
2	125			1				1	124
3	53	1	1		1			3	50
4	70			2	4	1		7	63
Total	376	6	3	4	5	1	1	20	356

IOP = intraocular pressure; RE = refractive error; VF = visual field.

Mean sensitivity (SD) was 29.1 (1.3) decibels (dB) for the 24-2 test pattern and 32.4 (1.0) dB for the 10-2 pattern. For the 24-2 and 10-2 test patterns, the results changed with age—with mean sensitivity decreasing by an average of 0.06 (0.01) dB per year and 0.05 (0.01) dB per year, respectively. The range of pointwise threshold sensitivity increased in size with eccentricity and exhibited asymmetry around the mean. The mean (SD) binocular test duration was 3.18 (0.38) minutes (1 minute 35 seconds per eye) for the 24-2 test and 3.58 (0.43) minutes (1 minute 47 seconds per eye) for the 10-2 test.

**Figure 2:**
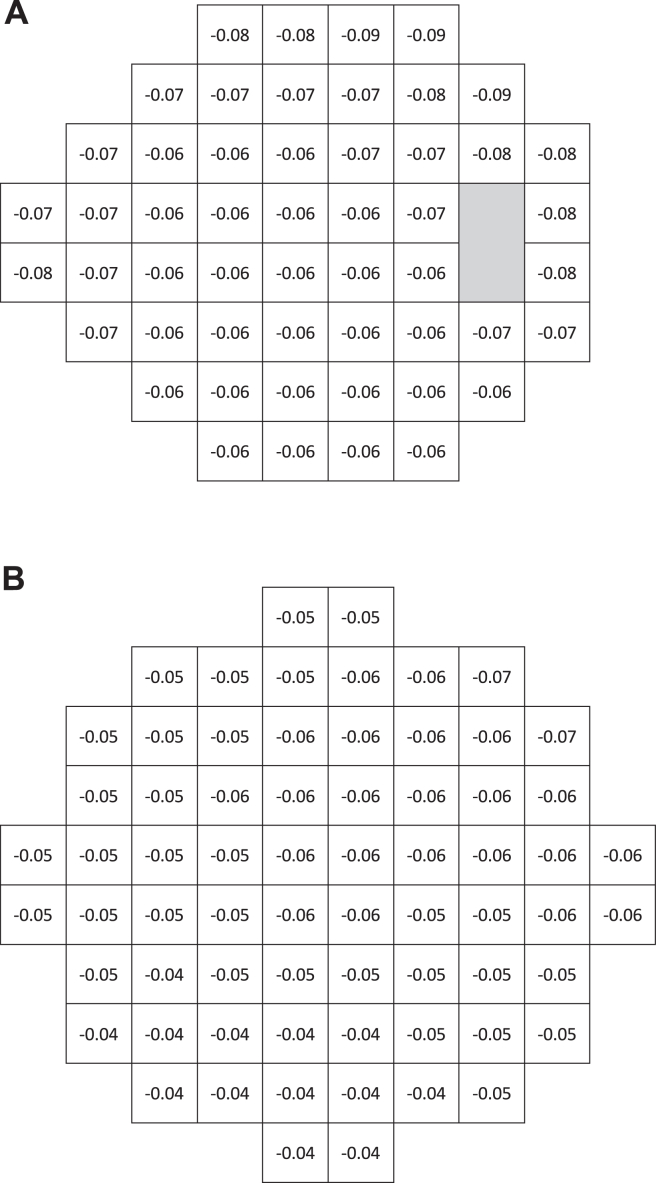
Pointwise cross-sectional age rate of decline in average sensitivity across the tested 24-degree (**A**) and 10-degree (**B**) fields of TEMPO.

Figure 2A presents the cross-sectional age rate of decline in sensitivity at each 24-2 test point, measured in dB per year of age. Overall, the rates in the superior, temporal, and nasal periphery tended to be slightly larger than rates found in the paracentral region. Similarly, Figure 2B presents the cross-sectional age rate of decline in sensitivity at each 10-2 test point, measured in dB per year. Overall, the sensitivity slopes in the inferior hemifield were lower than those in the superior hemifield.

**Figure 3:**
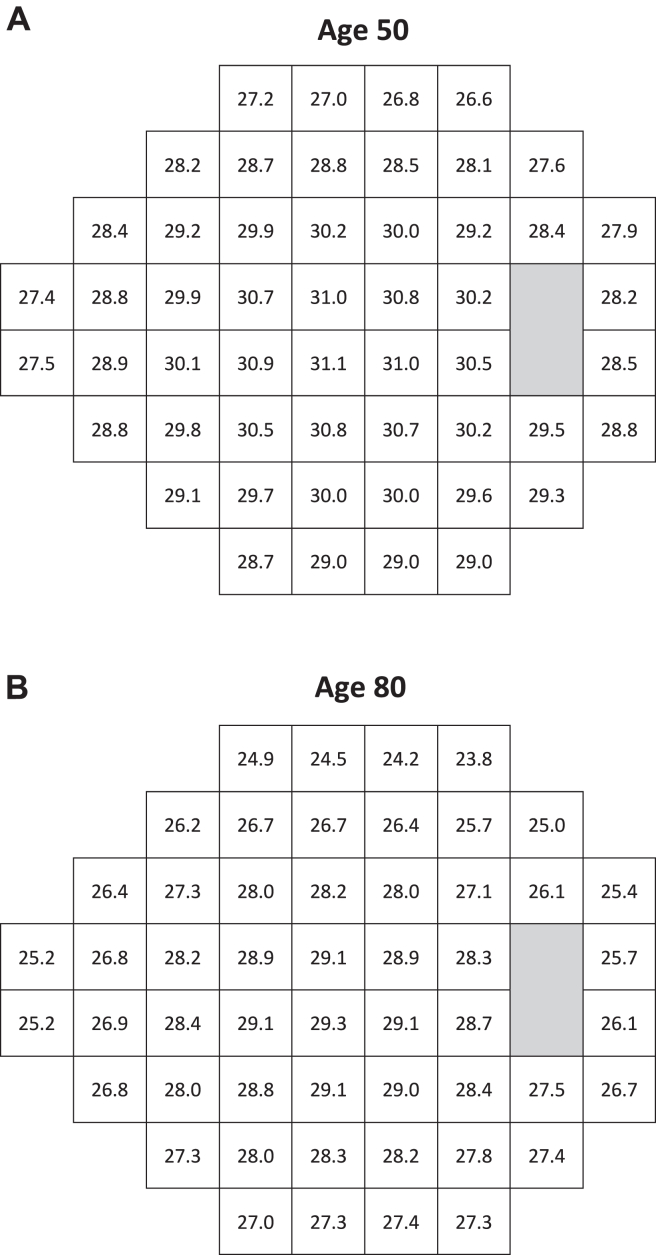
Pointwise age-corrected 50th percentile thresholds across the tested 24-degree field of TEMPO, calculated for 50-year-old (**A**) and 80-year-old subjects (**B**).

Age-corrected 50th percentile 24-2 threshold values are shown for 50- and 80-year-old subjects in Figure 3. As expected, the older subject exhibited more decline in sensitivities compared with the 50-year-old subject. These age-related differences in sensitivities reflect the rates of decline in sensitivity at each test point, as presented in Figure 2A.

**Figure 4:**
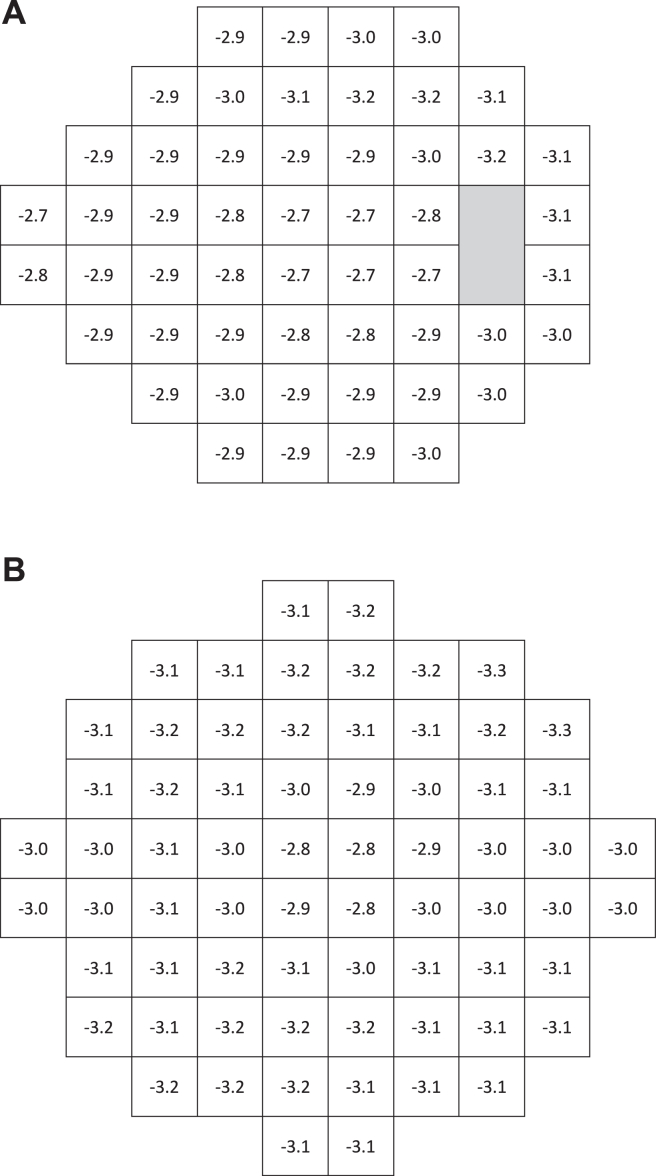
Pointwise *P* < 0.05 significance limits for decibel deviations from expected age-corrected sensitivities (total deviation) at each point in the 24-2 (**A**) and 10-2 (**B**) test patterns of TEMPO.

Figure 4A illustrates pointwise intersubject variation for 24-2, with *P* < 0.05 significance limits indicating deviations from expected age-normal sensitivities. Similarly, Figure 4B presents the corresponding 10-2 significance limits. Intersubject variation in both test patterns was lowest centrally and increased slightly with eccentricity, particularly in the temporal region of the 24-degree field (Fig 4A).

## Discussion

We have established an RDB for the TEMPO perimeter. Our results have qualitatively confirmed earlier findings obtained using other SAP perimeters regarding asymmetric age-related changes in the 24-2 hill of vision, in which peripheral test points exhibited larger aging changes compared with those at central test points (Fig 2A). ^5^^,^^18^ Age rates of decline for the 10-2 may appear to vary less with eccentricity than those from the 24-2, simply because 10-2 test points extend only 9 degrees from fixation. Thus, we did not expect to observe significant 10-2 spatial asymmetries in aging effects, and indeed, we did not (Fig 2B).

Figure 3 is based on the aging coefficients presented in Figure 2 and illustrates the expected magnitude of age effects over a 30-year period, during which sensitivity can decrease by as much as 2.5 dB in 24-2 tests.

The *P* < 0.05 significance limits for dB deviations from age-normal sensitivity (total deviation) at each test point are depicted for the 24-2 and 10-2 test patterns, respectively (Fig 4A, B). As expected, these findings resemble, but are not identical to, values obtained from another widely used automated perimeter operating under similar testing conditions; this is likely because of design differences in testing strategies.^11^ It is essential to note that, in general, test results from different SAP devices are best compared relative to their own respective RDBs.^19^ Our significance limits also support previous observations of increasing intersubject variability with eccentricity (Fig 4A, B).^4^ However, our limits do not increase as much with eccentricity as reported elsewhere.^4^^,^^5^^,^^11^ There may be ≥2 reasons for this discrepancy. First, early publications on this topic used the 30-2 test pattern. Compared with the 24-2 pattern used in this study, the additional peripheral test points of the 30-2 have been reported to have significantly higher variability from person to person than macular points^5^ and are also more likely to be affected by trial lens artifacts. Second, TEMPO lens correction optics have a diameter of 50 mm, which is considerably larger than the standard 38-mm trial lenses used in most perimeters, thus further reducing the likelihood of trial lens artifacts when using the TEMPO device.

The perimeter used in this trial is capable of simultaneously testing both eyes, and we speculated this would improve clinical efficiency. In this regard, the mean (SD) binocular test duration was 3.18 (0.38) minutes (1 minute 35 seconds per eye) for the 24-2 test, and only slightly longer for the 10-2.

This study has some limitations. First, testing of normal subjects was conducted at only 4 sites, all located in the United States. One might argue that testing at a larger number of sites might better capture any effects of intersite variations in clinical testing procedures. However, there also are advantages associated with limiting the number of test sites, particularly the importance of carefully training and monitoring all involved perimetric technicians. Thus, the study was designed to closely focus on maintaining accepted perimetric testing practices at only 4 trial locations.

Second, our study was conducted in clinical settings rather than being based on population-based samples. It is notable that ≥1 early perimetric RDBs relied partially on population-based data.^4^^,^^11^ In contrast, to the best of our knowledge, modern RDBs were sourced from university-based or private ophthalmology and optometry clinics; they utilized subjects who were free of diseases known to affect the VF. This methodology has been accepted by the United States Food and Drug Administration and other regulatory agencies. Each subject underwent an eye examination to exclude ocular abnormalities that would disqualify them from participation. Furthermore, 2 experienced clinicians independently reviewed all study VFs to exclude obvious testing artifacts and inspected color fundus photographs associated with any field test showing evident VF loss. Visual fields with defects that corresponded to fundus photo abnormalities were excluded from study. Additionally, subjects known to be highly experienced at performing VF testing were excluded. Third, the generalizability of our findings to ethnicities or races not commonly encountered in the United States should be considered. There is discrepancy in the literature regarding differences in visual function between healthy eyes of different ethnicities. For instance, Racette et al compared automated perimetry and imaging findings in healthy eyes of 50 self-identified Black subjects and 50 self-identified White subjects, revealing significant racial differences in ocular structure but no detectable differences in visual function as measured using SAP.^20^ Likewise, Sample et al found that among approximately 1250 subjects, half of African descent and the other half of European descent, there were no clinically significant racial variations observed in mean deviation and pattern SD findings.^21^ However, another study by the same research group found that healthy participants of African descent exhibited slight but statistically different performance, compared with participants of European descent.^22^ Nevertheless, future studies will evaluate the generalizability of our findings to various ethnicities and races.

Fourth, in this study, 15 subjects were unable to undergo binocular testing. Previous research has suggested that binocularly determined visual sensitivity is higher than monocular sensitivity at the fovea and within 5 degrees of fixation but becomes similar beyond 5 degrees. Moreover, monocular sensitivity without occlusion may be influenced differently by binocular interaction due to sensitivity disparity between the eyes.^23^^,^^24^ In our study, the majority of tested points were situated beyond 5 degrees. Additionally, differences in monocular versus binocular sensitivity might be more pronounced in eyes with pathology, compared with subjects with normal vision. Therefore, future studies should be conducted to test eyes with various VF defects, exploring whether disparities between sensitivities with and without occlusion vary based on the severity, location, and unilaterality/bilaterality of the defects.

Testing the diagnostic accuracy of the TEMPO perimeter, as well as comparing its VF test measurements to other forms of SAP, are important subjects for upcoming studies.

In summary, we tested 356 ocularly healthy subjects with a new perimeter and produced an RDB for 24-2 and 10-2 test patterns.
