# Supplementary material for: Reference Database for a Novel Binocular Visual Function Perimeter: A Randomized Clinical Trial
Source: Ophthalmol Sci. 2024 Jul 20;4(6):100583. doi: 10.1016/j.xops.2024.100583 (PMC11388689; doi:10.1016/j.xops.2024.100583)

# Flow Diagram

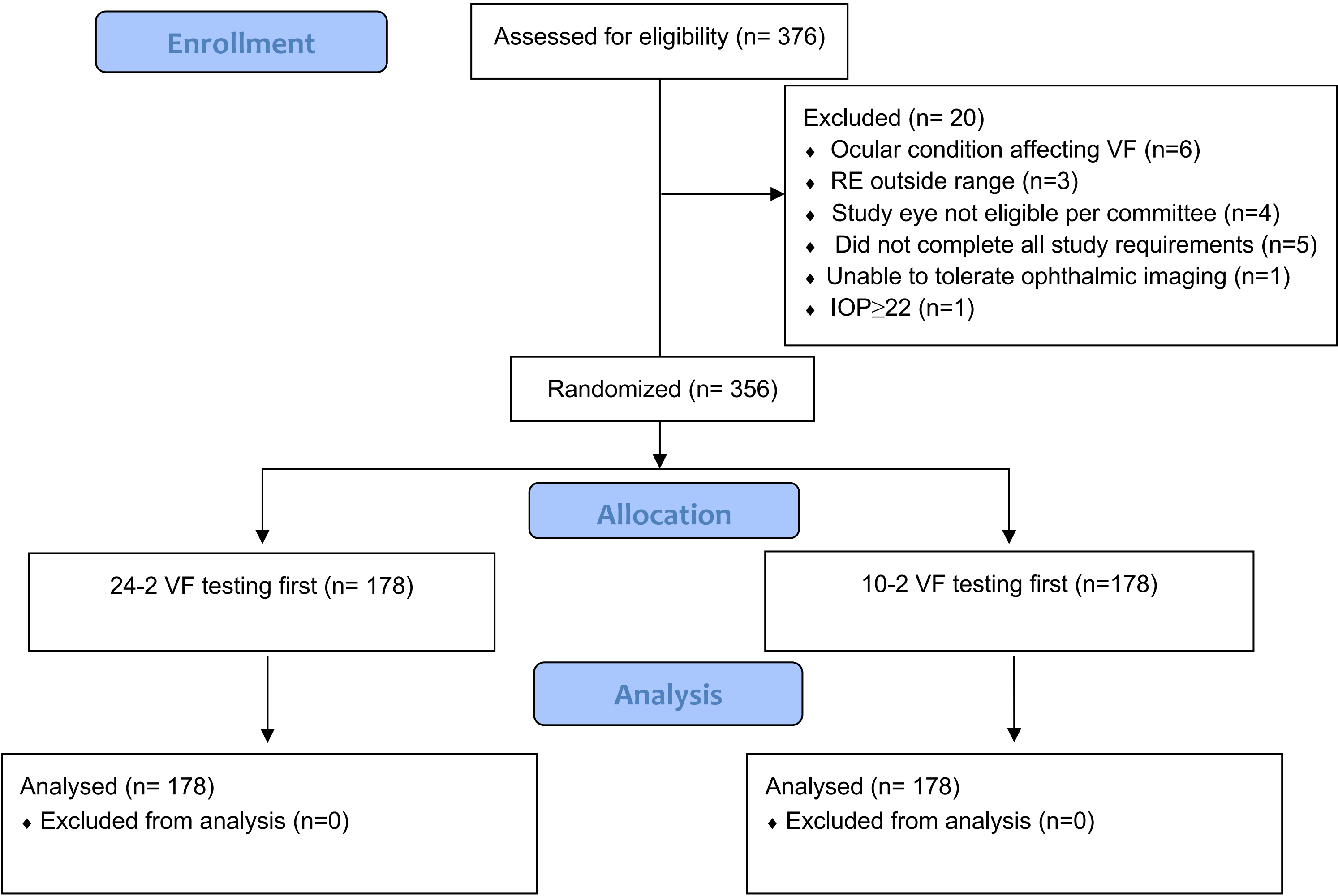

Supplement: Figure S1 [file mmc1.pdf]
